# Overall hemostatic potential as a marker of subclinical hypercoagulability in treated psoriasis patients

**DOI:** 10.3389/fmed.2025.1611827

**Published:** 2025-08-21

**Authors:** Eva Klara Merzel Šabović, Tadeja Kraner Šumenjak, Mojca Božič Mijovski, Miodrag Janić

**Affiliations:** ^1^Department of Dermatovenerology, University Medical Centre Ljubljana, Ljubljana, Slovenia; ^2^Faculty of Medicine, University of Ljubljana, Ljubljana, Slovenia; ^3^Faculty of Agriculture and Life Sciences, University of Maribor, Hoče, Slovenia; ^4^Department of Vascular Diseases, Laboratory for Haemostasis and Atherothrombosis, University Medical Centre Ljubljana, Ljubljana, Slovenia; ^5^Clinical Department of Endocrinology, Diabetes and Metabolic Diseases, University Medical Centre Ljubljana, Ljubljana, Slovenia

**Keywords:** psoriasis, hypercoagulability, overall hemostatic potential (OHP), fibrinolysis, obesity

## Abstract

**Background:**

Psoriasis is associated with increased cardiovascular risk, possibly mediated by inflammation-induced hemostatic dysregulation and hypercoagulability. However, these changes are often difficult to detect with conventional markers.

**Objectives:**

To assess hypercoagulability in patients with psoriasis using the Overall Hemostatic Potential (OHP) test, a global integrative test for coagulation and fibrinolysis.

**Methods:**

We studied 80 psoriasis patients (54 men, 26 women, aged 30–45 years) receiving effective topical or systemic treatments (methotrexate, adalimumab, secukinumab or guselkumab) and compared them with 20 healthy controls. We measured OHP, its components — overall coagulation potential (OCP) and overall fibrinolytic potential (OFP) and selected hemostatic markers (platelet count, mean platelet volume, platelet-to-lymphocyte ratio, P-selectin, D-dimer and fibrinogen).

**Results:**

Psoriasis patients had significantly higher OHP levels, primarily due to decreased OFP, while OCP levels were comparable to the control group — indicating a hypercoagulable state due to impaired fibrinolysis. Other conventional hemostatic markers showed no significant differences. OHP and OFP correlated with residual inflammatory activity, BMI, waist circumference, visceral adiposity and fibrinogen levels, suggesting a relationship between subclinical inflammation, metabolic parameters and hemostatic imbalance.

**Conclusion:**

The OHP test reveals a hypercoagulable state in psoriasis patients even in the absence of abnormal standard coagulation markers. OHP could be a practical and sensitive tool to stratify cardiovascular risk in psoriasis, especially in patients with concomitant metabolic disease or persistent inflammation.

## Graphical abstract

**Figure:**
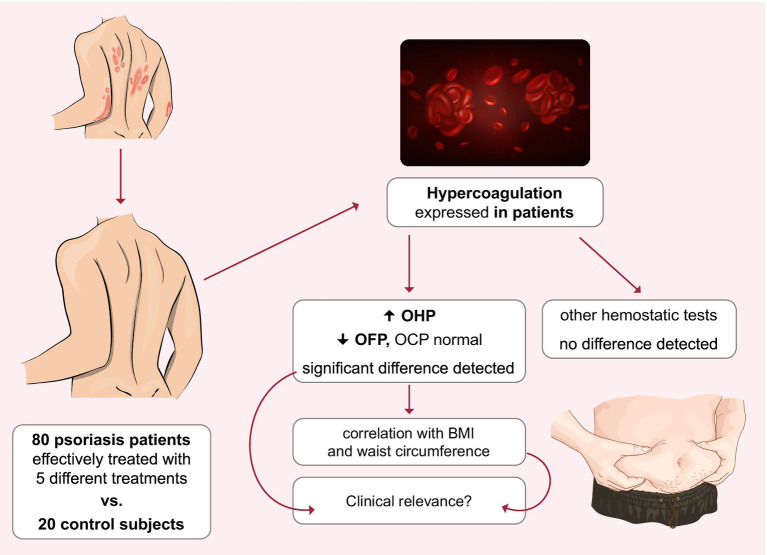
Hypercoagulability measured by OHP but not by hemostatic markers was present in psoriasis patients despite effective treatment. This hypercoagulability correlated with body mass index and waist circumference. This correlation was not observed in control subjects. OHP, overall hemostatic potential; OCP, overall coagulation potential; OFP, overall fibrinolytic potential; BMI, body mass index.

## 1 Introduction

Psoriasis is a chronic immune-mediated disease that affects 2–3% of the population (1). It is associated with several comorbidities, with cardiovascular disease being the major cause of morbidity and mortality (1–3). While the exact mechanisms of increased cardiovascular risk are still unclear, it is known that systemic inflammation in psoriasis contributes to endothelial dysfunction and the atherosclerosis process (3–5). It also leads to hypercoagulability, a potential risk factor for both arterial and venous thrombosis (4, 6, 7). Psoriasis patients frequently suffer from arterial thrombotic events such as myocardial infarction and stroke and venous thrombotic events such as venous thromboembolism (8–10). Several questions about possible hypercoagulability in psoriasis patients remain unanswered.

Current treatments for psoriasis can achieve complete or near-complete clearance of skin lesions in most cases, and studies have shown that this improvement is often accompanied by a significant reduction in systemic inflammation (11, 12). Given the link between inflammation and hypercoagulability in psoriasis, it could be hypothesized that reducing systemic inflammation through effective treatment could also eliminate hypercoagulability. Hypercoagulability has been shown to be present in patients with psoriasis compared to healthy controls (9), but there is a paucity of studies investigating whether hemostatic impairment is also present in patients successfully treated with antipsoriatic drugs, particularly biologics. This question is further complicated by the problems with laboratory measurement of hemostasis, as specific tests may not capture all aspects of hypercoagulability and therefore may not detect it. A broader, non-specific and more sensitive test to detect impaired hemostasis appears to be needed to address this issue.

The aim of the present study was to investigate whether hemostasis is activated in effectively treated patients with psoriasis. For this purpose, the overall hemostatic potential (OHP) test was used, whose subcomponents are the overall fibrinolytic potential (OFP) and the overall coagulation potential (OCP) (13). OHP is a non-specific, global, broad-based test for hemostasis that has been shown to be very sensitive in detecting imbalances in hemostasis and explaining which part of hemostasis, either fibrinolysis or coagulation, is altered (14). OHP has not yet been used in the study of psoriasis patients. We are investigating hemostasis with OHP in effectively treated psoriasis patients, as this group is very large and obviously of particular interest due to current effective treatment. We hypothesized that complete clearance of the skin lesions would completely eliminate possible inflammation-induced hypercoagulability, and that hemostasis profile would be comparable to that of control subjects. In addition, we investigated the factors that could be associated with possible hypercoagulability.

## 2 Materials and methods

### 2.1 Study population and design

A cross-sectional study was conducted at the Dermatovenerology clinic, University Medical Centre Ljubljana, Ljubljana, Slovenia. A total of 80 psoriasis patients (54 men and 26 women) were consecutively recruited between March 2022 and December 2023. Twenty healthy subjects (11 men and 9 women) served as controls. The patients were effectively treated with (1) topical therapy (*n* = 21), (2) methotrexate (*n* = 11), (3) adalimumab (*n* = 14), (4) secukinumab (*n* = 14), and (5) guselkumab (*n* = 20). Treatment efficacy was defined by the Psoriasis Area Severity Score (PASI). 99% of patients achieved an excellent response (PASI < 1), while 1% achieved a good response (PASI 1–5). Both the patients and the physician had to be satisfied with the response to treatment without the intention of changing it. The inclusion criteria were a diagnosis of psoriasis, age between 30 and 45 years, effective treatment of psoriasis, stable clinical course with a consistent PASI score for at least 6 months. Exclusion criteria were previous cardiovascular events, type 1 or type 2 diabetes, psoriatic arthritis or other chronic inflammatory diseases, menopause, pregnancy or breastfeeding, and other pharmacologic treatments in addition to psoriasis therapy. Healthy control subjects aged 30 to 45 years were selected according to the same exclusion criteria. All participants voluntarily participated in this study and gave their informed written consent. The study is registered at http://clinicaltrials.gov (ClinicalTrials.gov Identifier: NCT05957120) and is in accordance with the STROBE guidelines (15).

### 2.2 Study protocol

During the study appointment, a complete medical history was obtained from each study participant, and a complete medical examination was performed, including duration of psoriasis, duration of treatment and smoking status. Each participant’s anthropometric measurements (weight, height and waist circumference), systolic and diastolic blood pressure and heart rate were determined. In addition to the other measurements, fasting blood samples were taken from each participant by venipuncture according to the standard procedure.

### 2.3 Laboratory methods

Fasting blood samples were collected in a 4.5 mL clotting tube containing 0.109 mol/L sodium citrate, a 4 mL vacuum tube containing clot activator, and a 2 mL K2-EDTA tube. A complete differential blood count was obtained from fresh EDTA blood using the XN-1000 hematology analyzer (Sysmex, Japan) to determine platelet count, mean platelet volume (MPV), and platelet-to-lymphocyte ratio (PLR). Serum and plasma were prepared by centrifugation at 2,000 × g for 20 min. Immediately after centrifugation, serum and plasma were aliquoted and stored at ≤ −70°C until analysis. P-selectin concentration in serum was measured with xMAP© technology using magnetic beads coupled with specific antibodies (all R&D Systems, USA) on a MagPix device (Luminex Corporation, USA). D-dimer and fibrinogen concentrations were measured in plasma with a CS-5100 automated coagulation analyzer (Sysmex, Japan) using the Innovance D-dimer reagent kit and Dade Thrombin Reagent, respectively (both Siemens Healthineers, Germany).

OHP (abs-sum), OCP (abs-sum) and OFP (%) were determined as previously described (16) using bovine thrombin (Sigma Chemical Company, USA) and the recombinant tissue-type plasminogen activator (Actilyse 0.1 mg/mL, Boehringer Ingelheim, Germany) by absorbance measurements at 405 nm at 1-min intervals over 40 min, as previously described (13). The curve ranges were constructed for OHP and OCP using the measurements obtained; OFP was calculated as the relative difference between the above two ranges as OFP = (OHP – OCP)/OCP × 100%.

### 2.4 Statistical analysis

The statistical analyses were performed using R (version 4.2.2) and IBM SPSS Statistics 28. Due to the non-normal distributions and the presence of outliers, patient characteristics were presented as group medians with interquartile ranges. The non-parametric Kruskal-Wallis test was used to test the null hypothesis of equal population medians. The Fisher–Freeman–Halton exact test was performed to assess the association between two categorical variables. To examine the effects of psoriasis treatment type, smoking status (smokers vs. non-smokers), and body mass index (BMI ≤ 25 vs. > 25), we applied Quade’s nonparametric three-way ANCOVA, with systolic blood pressure and age included as covariates. Quade’s method was chosen as it allows adjustment for covariates while comparing multiple factors in datasets that do not meet parametric assumptions.« For significant results, Bonferroni adjusted pairwise comparisons were used to control multiple comparisons.

We have performed a comprehensive correlation analysis. For the correlation matrix, we used both the reported parameters, and a large set of parameters reported in our previous articles examining the same patient and control cohort (17, 18). In the analysis we included markers for metabolic dysregulation cholesterol, LDL cholesterol, triglycerides, triglyceride-glucose index (TyG), homeostatic model assessment for insulin resistance (HOMA-IR), fibrosis-4 index (FIB-4) (18) and C-reactive protein (CRP) as a marker of inflammation (19).

## 3 Results

The main characteristics of the patients are listed in Table 1. Of the included patients, 37% were smokers, unevenly distributed between treatment groups, and 65% had a body mass index (BMI) ≥ 25.0 and waist circumference (≥ 94 cm in men and ≥ 80 cm in women), while in the control group 30% were smokers and 40% had a high BMI and waist circumference.

**Table 1:** Characteristics of patients.

Patients’ characteristics		TOP (*n* = 21)	MTX (*n* = 11)	ADA (*n* = 14)	SEC (*n* = 14)	GUS (*n* = 20)	CG (*n* = 20)	Test statistics *p*-value
Average age (years)		38.00 (32.00–41.50)	39.00 (35.00–42.00)	39.50 (36.75–41.00)	39.50 (34.50–43.25)	40.00 (36.00–43.00)	34.50 (31.25–39.75)	H = 9.312 *p* = 0.097
Sex	Male	13	7	10	11	12	13	Exact test *p* = 0.905
	Female	8	4	4	3	8	7	
PASI		0.2 (0.1–1.75)	1.2 (0.7–3.2)	0.0 (0.0–0.2)	0.0 (0.0–0.65)	0.0 (0.0–0.6)	/	H = 17.393 *p* = 0.002
BSA (m^2^)		1.0 (1.0–1.5)	2.0 (1.0–4.0)	0.0 (0.0–0.25)	0.0 (0.0–1.0)	0.0 (0.0–1.0)	/	H = 20.849 *p* < 0.001
Duration of psoriasis (years)		8.0 (4.5–20.0)	10.0 (5.0–12.0)	20.0 (11.5–25.0)	16.5 (13.75–23.25)	20.0 (15.0–23.5)	/	H = 16.646 *p* = 0.002
Duration of treatment (months)		77.0 (47.0–239.0)	29.0 (21.0–47.0)	95.0 (61.0–117.5)	48.0 (33.5–59.0)	31.0 (27.3–51.0)	/	H = 31.059 *p* < 0.001
BMI (kg/m^2^)		23.36 (22.59–26.18)	28.05 (22.65–37.51)	27.04 (23.62–30.66)	31.04 (26.75–35.39)	27.50 (24.54–34.96)	24.30 (23.40–26.75)	H = 17.766 *p* = 0.003
Waist circumference (cm)		86.00 (77.50–94.50)	104.00 (92.00–121.50)	93.25 (89.125–109.375)	104.50 (98.00–113.50)	99.25 (85.625–108.875)	90.00 (79.75–94.00)	H = 24.767 *p* < 0.001

Data are presented as the median (interquartile range), or number of cases for categorical variable. The *p*-value in the last column was determined using the Kruskal-Wallis H-test or Fisher–Freeman–Halton exact test. TOP, topical therapy; MTX, methotrexate; ADA, adalimumab; SEC, secukinumab; GUS, guselkumab; CG, control group; PASI, Psoriasis Area and Severity Index; BSA, Body Surface Area; BMI, body mass index.

**Figure 1:**
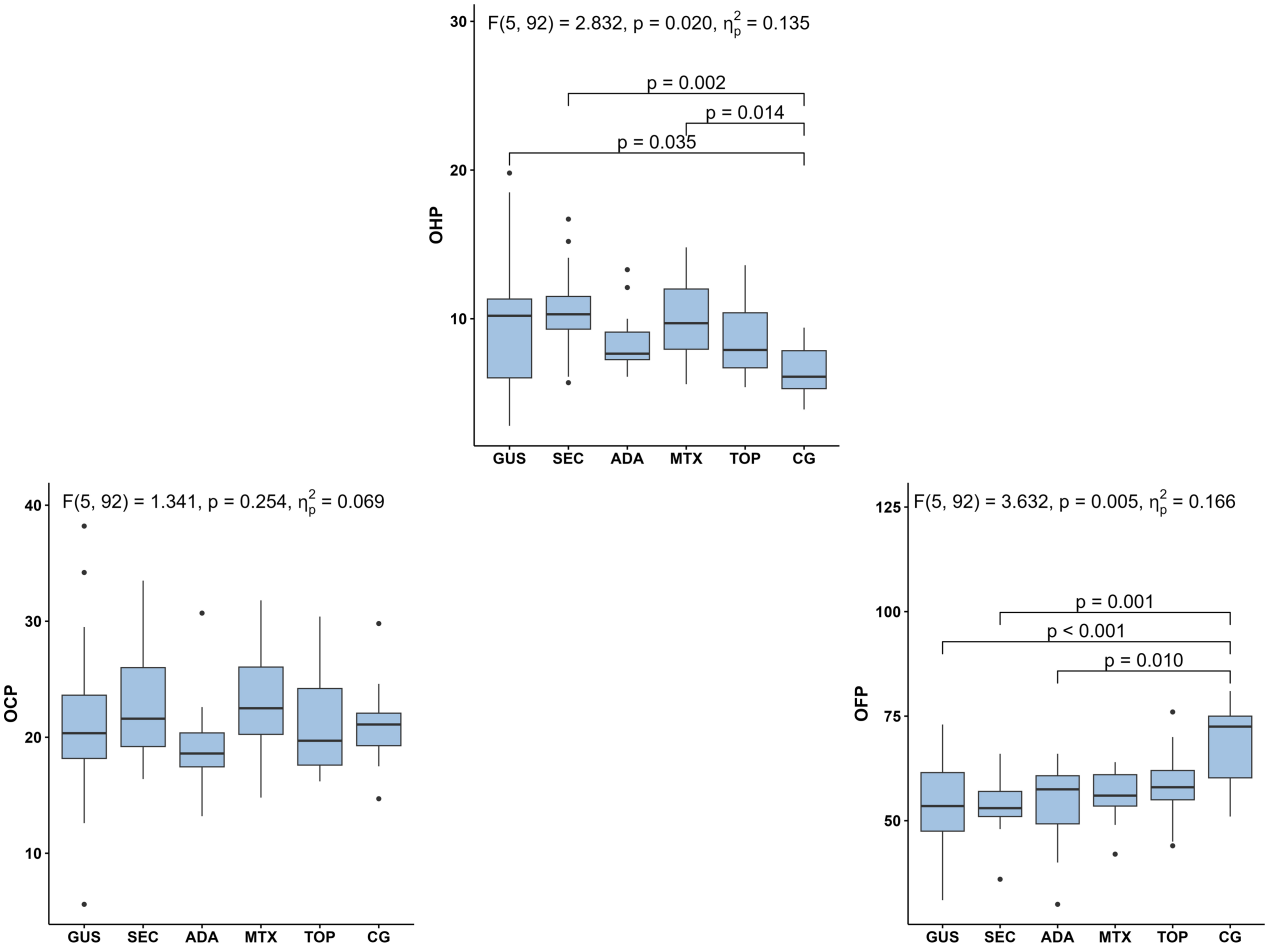
Selected markers of hemostasis (OHP, OCP, OFP) in five psoriasis treatment groups and controls. Group differences were analyzed using Quade’s ANCOVA adjusting for age and systolic blood pressure, followed by Bonferroni-adjusted pairwise comparisons; only statistically significant differences (*p* ≤ 0.05) are shown. OHP, overall hemostatic potential; OCP, overall coagulation potential; OFP, overall fibrinolytic potential; GUS, guselkumab; SEC, secukinumab; ADA, adalimumab; MTX, methotrexate; TOP, topical therapy; CG, control group.

The results of OHP, OFP in OCP are shown in Figure 1. The entire group of psoriasis patients had higher OHP values and significantly lower OFP values compared to controls, while OCP values were similar in patients and controls. When analyzing separate groups of psoriasis patients with different treatment modalities, significantly higher OHP levels were found for the guselkumab (*p* < 0.05), secukinumab (*p* < 0.01) and methotrexate (*p* < 0.05) treated groups compared to controls, while little significant difference was found in the topically treated groups (*p* = 0.08). Significantly lower OFP levels were found in the groups treated with guselkumab (*p* < 0.01), secukinumab (*p* < 0.01) and adalimumab (*p* < 0.05) compared to the controls. In contrast to OHP and OFP, no significant differences were found in OCP levels between treatments.

**Figure 2:**
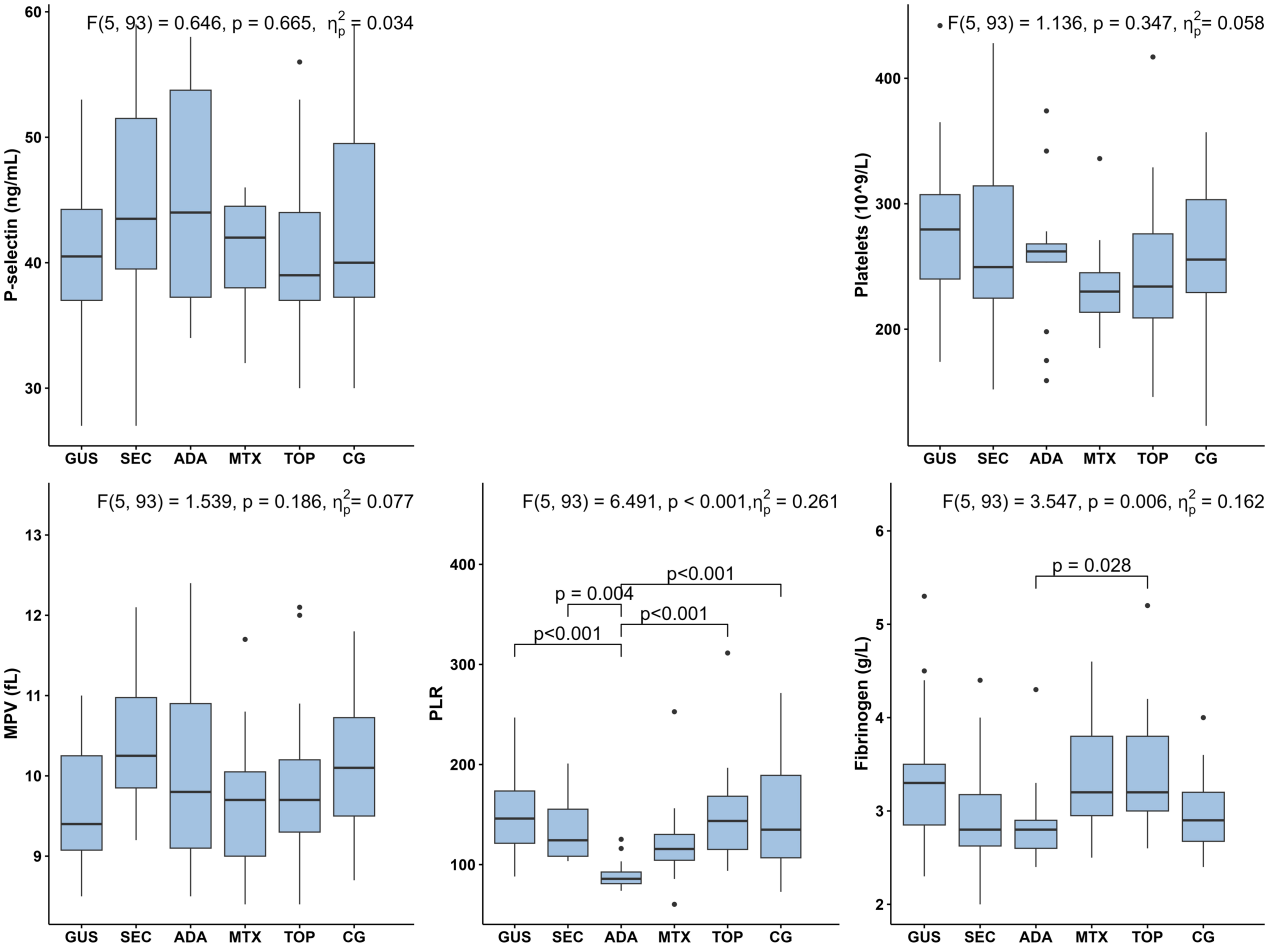
Selected markers of hemostasis (P-selectin, platelets, MPV, PLR, fibrinogen) in five psoriasis treatment groups and controls. Group differences were assessed using Quade’s ANCOVA adjusting for age and systolic blood pressure, followed by Bonferroni-adjusted pairwise comparisons; only statistically significant differences (*p* ≤ 0.05) are shown. MPV, mean platelet volume; PLR, platelet-to-lymphocyte ratio GUS, guselkumab; SEC, secukinumab; ADA, adalimumab; MTX, methotrexate; TOP, topical therapy; CG, control group.

**Figure 3:**
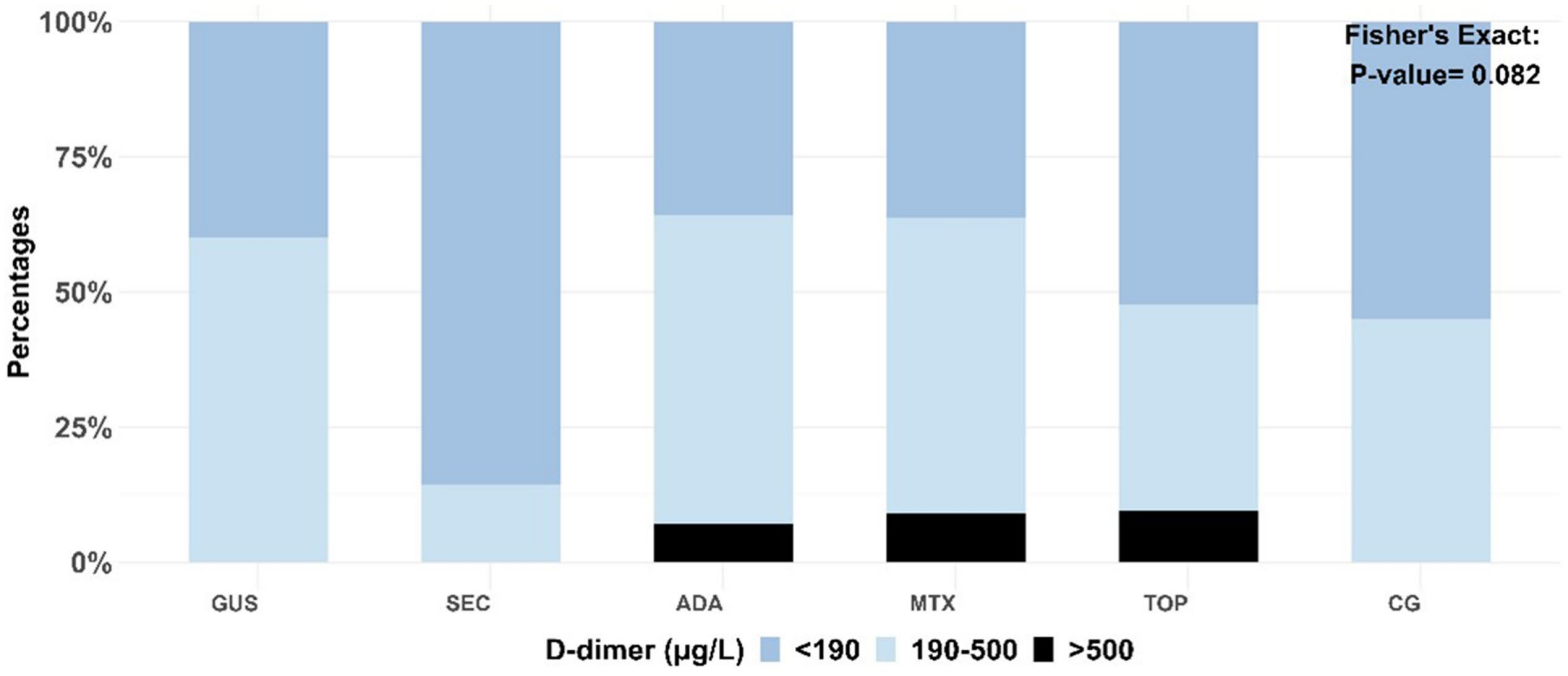
Distribution of D-dimer levels by treatment type. The association between categorical variables was measured using Fisher’s exact test. GUS, guselkumab; SEC, secukinumab; ADA, adalimumab; MTX, methotrexate; TOP, topical therapy; CG, control group.

Other hemostasis markers are shown in Figure 2. Platelet count, platelet activation markers (MPV, PLR), P-selectin and fibrinogen are shown in Figure 2 and D-dimer in Figure 3. The results showed no statistical differences in platelet count, MPV, P-selectin and D-dimer between the entire patient group, and the individual patient and control groups. Psoriasis patients treated with adalimumab had a significantly lower median PLR compared to all other groups except the methotrexate-treated group. Fibrinogen levels were significantly lower in the adalimumab-treated group than in the topical treatment group. Otherwise, there were no significant differences.

Body mass index (BMI) showed a significant association with both OHP and P-selectin levels. Participants with BMI > 25 had a higher median OHP (9.9) compared to those with BMI ≤ 25 (7.4). Likewise, P-selectin levels were higher in the elevated BMI group (median 43 vs. 39), suggesting a link between increased body weight and hemostatic changes. Regarding smoking, smokers had significantly higher P-selectin levels (median 45) compared to non-smokers (median 39). These findings confirm the influence of metabolic factors and smoking on certain coagulation parameters within our cohort.

We performed the correlation analysis between hemostasis markers, patient demographics and anthropometric measures. OHP showed a positive correlation with BMI (*r* = 0.486, *p* < 0.001) and waist circumference (*r* = 0.446, *p* < 0.001) and a positive correlation with OCP (*r* = 0.642, *p* < 0.001) and fibrinogen (*r* = 0.616, *p* < 0.001). Conversely, OFP showed a negative correlation with BMI (*r* = −0.506, *p* < 0.001), waist circumference (*r* = −0.503, *p* < 0.001) and a negative correlation with OHP (*r* = −0.710, *p* < 0.001). A positive correlation was found between OCP and fibrinogen (*r* = 0.740, *p* < 0.001).

In the next correlation analysis, OHP, OFP and OCP levels were analyzed with respect to markers of residual inflammation and metabolic dysregulation associated with visceral obesity. OHP correlated with HOMA-IR (*r* = 0.441, *p* < 0.001) and CRP (*r* = 0.638, *p* < 0.001). OCP correlated only with CRP (*r* = 0.581, *p* < 0.001). The most significant correlations were found for OFP, which correlated negatively with triglycerides (*r* = −0.465, *p* < 0.001), TyG (*r* = −0.497, *p* < 0.001), HOMA-IR (*r* = −0.553, *p* < 0.001) and CRP (*r* = −0.408, *p* < 0.001). On the other hand, OHP and OFP did not correlate with markers of metabolic dysregulation or CRP in the control group.

## 4 Discussion

The hemostatic imbalance in psoriasis patients is not yet fully understood. Using the OHP test, the global integrated test for blood coagulation, we found that hypercoagulability was present in psoriasis patients effectively treated with five different therapies. OHP was associated with decreased OFP, suggesting decreased fibrinolytic activity as the main cause of hypercoagulability. Increased OHP was found in psoriasis patients regardless of disease severity, both in those treated with topical therapy alone and in those treated with systemic therapy, including three different biologics. OHP and OFP correlated with residual inflammation (CRP) and BMI, waist circumference and markers of metabolic dysregulation associated with visceral obesity in psoriasis patients, but not in controls. Further studies are needed to clarify the clinical value of the observed hypercoagulability and to explain the role of residual inflammation and overweight/obesity on hypercoagulability and cardiovascular risk in effectively treated psoriasis patients.

Although the presence of hypercoagulability in psoriasis patients has been documented, there is insufficient data on possible hypercoagulability in effectively treated psoriasis patients (9). It could be assumed that hypercoagulability is eliminated by the clearance of skin lesions and inflammatory foci. The study of hemostasis in psoriasis is complicated by the fact that currently widely used hemostatic tests are specific rather than global. To overcome this obstacle, we used the OHP test, a global integrated test that measures the dynamics of fibrin formation and degradation in plasma (13, 14). It generates a fibrin time curve using optical density measurements that reflect the interplay between clotting driven by thrombin or tissue factors and fibrinolysis initiated by tissue plasminogen activator. OHP quantifies both procoagulant (OCP) and fibrinolytic (OFP) activity by assessing the combined effect of procoagulant, anticoagulant and fibrinolytic factors in plasma (14). OHP is particularly useful in detecting hypercoagulable states that may be missed by standard hemostasis tests (13, 14). It has been successfully used to detect hemostatic abnormalities in various conditions such as coronary artery disease, stroke, deep vein thrombosis, diabetes, pregnancy, chronic kidney disease, carcinoma, and others (13, 14). Its simplicity and cost make it a valuable tool for the assessment of hemostatic abnormalities. OHP has not yet been used in psoriasis patients. Compared to the selected hemostatic tests used in our study, related to either procoagulant or anticoagulant activity, OHP showed a higher sensitivity for the detection of global hemostatic abnormalities. It is likely that the broader, nonspecific nature of OHP, representing a broad combination of procoagulant, anticoagulant, and fibrinolytic factors, is an important advantage of OHP. Overall, our results have shown that OHP appears to be a suitable test for the detection of hypercoagulability in psoriasis patients.

Psoriasis treatments may differentially affect systemic hemostasis through their distinct mechanisms of action. Topical therapy mainly reduces local inflammation, with limited systemic impact on coagulation pathways. Methotrexate, due to its systemic anti-inflammatory effects, may attenuate endothelial activation and thrombotic risk. Biologic agents—anti-TNF (adalimumab), anti-IL-17A (secukinumab), and anti-IL-23 (guselkumab)—target key inflammatory cytokines involved in vascular inflammation, coagulation, and fibrinolysis. Although available clinical data are limited, these mechanistic differences could underline the possible variability in global hemostatic parameters across treatment groups.

We found that increased OHP was associated with decreased OFP, suggesting decreased fibrinolytic activity rather than increased coagulation activity in psoriasis patients. Thus, the balance between coagulation and fibrinolysis is shifted in favor of coagulation by reduced fibrinolytic activity. This observation is important for the study of the mechanisms of hemostasis impairment in psoriasis patients. Interestingly, both OHP and OFP correlated with BMI and waist circumference in psoriasis patients but not in control subjects. By extending the correlation matrix with data from previous studies in the same patient and control cohort (17, 18, 20) to include markers of metabolic dysfunction and inflammation, a significant correlation was found with markers of metabolic dysfunction associated with visceral obesity (triglyceride levels, TyG index and HOMA-IR), as well as with CRP as an inflammatory marker. In controls, there was no correlation between OHP and OFP on one side and markers of metabolic dysfunction and CRP on the other. Our results show that psoriasis patients who have the most pronounced metabolic dysregulation associated with (visceral) obesity also have the most pronounced hypercoagulability. It is already known that (visceral) obesity can influence hypercoagulability to a certain degree (21), but in psoriasis this association seems to be even more pronounced.

The bidirectional relationship between obesity and psoriasis is well known, less well known is the role of overweight/obesity in abnormal hemostasis. Obesity has been shown to be associated with impaired hemostasis (22). Extending this observation, our results show that in psoriasis patients, increased BMI and waist circumference impair hemostasis even more, as we found no correlation between OHP and OFP with BMI or waist circumference in controls. This suggests that psoriasis-induced inflammation and excess adipose tissue-induced inflammation may reinforce each other and lead to a more pronounced prothrombotic state. It could be hypothesized that visceral adiposity in psoriasis is in some way different than in individuals without psoriasis, such that visceral adiposity is more active in pathological metabolism, leading to dysregulation of metabolism and downstream facilitated activation of hypercoagulability. If this is the case, treatment of overweight/obesity in psoriasis patients could be proposed to reduce this prothrombotic burden. Thus, non-pharmacologic and pharmacologic interventions targeting visceral obesity could reduce the increased cardiovascular prothrombotic risk as well as metabolic risk in psoriasis patients. This assumption remains to be tested.

Our analysis confirmed that both elevated BMI and smoking influence selected coagulation parameters within our cohort. Higher BMI was associated with increased OHP and P-selectin levels, while smoking correlated with elevated P-selectin. These findings suggest that metabolic factors—particularly obesity—alongside psoriasis-related inflammation and treatment, may jointly contribute to a prothrombotic state in psoriasis patients. This underscores the complex interplay between inflammation, metabolic status, and hemostasis, highlighting the need for further research in larger, stratified studies.

To date, the OHP assay has not been applied in patients with psoriasis. Based on our findings, OHP could become a valuable biomarker in this population for two key purposes: first, to detect abnormalities in hemostasis that contribute to the elevated cardiovascular risk observed in psoriasis, and second, to assess the effectiveness of urgently needed interventions, such as reducing residual inflammation and addressing the dysmetabolic burden linked to overweight and obesity. The OHP test and its components—the OCP and OFP—may serve as sensitive tools to reveal subclinical hypercoagulability not identified by conventional coagulation assays. This could facilitate earlier detection of patients at increased cardiovascular risk. In clinical practice, incorporating these assays could enhance current risk stratification strategies, enabling more personalized preventive approaches, such as intensifying anti-inflammatory treatment or considering anticoagulant prophylaxis in selected high-risk individuals. Nonetheless, longitudinal studies are necessary to validate these markers for routine clinical application and to establish evidence-based guidelines.

It is well known that inflammation and abnormal hemostasis are linked (22). Under normal circumstances, these two processes are carefully regulated. However, in chronic inflammatory diseases, pro-inflammatory cytokines drive the constant activation of the coagulation cascade, and coagulation can perpetuate inflammation (23). Coagulation and inflammation have been shown to be linked through key hemostatic molecules such as tissue factor, thrombin, the protein C system and the fibrinolytic pathway (22). This relationship between inflammation and coagulation may not only be important for vascular thrombotic diseases but may also have implications for microvascular impairments that cause organ damage (24). In addition, activated coagulation factors can also trigger an inflammatory response by inducing the production of cytokines in immune cells (25). It seems that residual inflammation in successfully treated psoriasis patients should also be a target for new interventions.

Our study has several limitations. One is the relatively small number of participants in each patient group, although power calculations showed that the sample size was sufficient to detect differences in OHP values. In addition, ideally only non-smoking and non-overweight/obese patients should be included to exclude confounding factors. However, to reflect the typical psoriasis population, in which smoking and overweight/obesity are prevalent, smokers and overweight patients were included in the study. The prevalence of smoking in psoriasis patients is estimated to be 20–30% (26), and our study is consistent with this, as 37% of participants smoked and 65% were overweight. The control group also consisted of 30% smokers and 40% overweight individuals. This inclusion provides realistic information for clinicians who should encourage patients with psoriasis to change their lifestyle. Given the known influence of smoking on the prothrombotic state, we included smoking status as a factor in the model; however, it was found to have no significant effect on any of the selected parameters, except for P-selectin. Another limitation is the lack of longitudinal data to assess the hemostatic profile over time and to track the cardiovascular events. We analyzed the biologic treatment groups separately, as their distinct targets—anti-TNF, anti-IL-17A, or anti-IL-23—may differentially influence hemostasis. Given the exploratory nature of our study and the subtle hemostatic changes observed, combining the groups could have obscured potential trends or relevant differences. Although the limited sample size constrains the robustness of conclusions, we believe that separate analyses better preserve potential insights and ensure a more transparent interpretation of the data. Importantly, we did not assess clinical cardiovascular outcomes; therefore, no direct conclusions can be drawn regarding the predictive value of OHP, OFP, or OCP for future cardiovascular events. Despite these limitations, we believe that our results provide important clinical insights into the hemostatic profile of effectively treated psoriasis patients.

In conclusion, our study shows that psoriasis patients have an abnormal hemostatic profile with hypercoagulability even when effectively treated with five different therapeutic modalities. Hypercoagulability detected by OHP and OFP correlated significantly with BMI, waist circumference, and markers of metabolic dysregulation and residual inflammation — associations that were not observed in healthy controls. These results suggest that effective psoriasis treatment alone cannot normalize hemostatic imbalance, especially in overweight or obese patients. Visceral obesity may represent an important therapeutic target to reduce hypercoagulability and cardiovascular risk in this population. OHP appears to be a promising marker for the identification of abnormal hemostasis in psoriasis, which warrants further clinical investigation.

## Data Availability

The raw data supporting the conclusions of this article will be made available by the authors, without undue reservation.
